# Slotted E-Shaped Meta-Material Decoupling Slab for Densely Packed MIMO Antenna Arrays

**DOI:** 10.3390/mi12080873

**Published:** 2021-07-25

**Authors:** Karim H. Moussa, Ahmed S. I. Amar, Mohamed Mabrouk, Heba G. Mohamed

**Affiliations:** 1 Electrical Department, College of Engineering, Horus University Egypt, New Damietta 34518, Egypt; Khassan@horus.edu.eg; 2 Department of Electronics and Communications Engineering, Ain Shams University, Cairo 11566, Egypt; ahmed.s.i.amar@ieee.org; 3 Electrical Engineering Department, Alexandria University, Alexandria 21421, Egypt; mouklly@yahoo.ca; 4 Electrical Department, College of Engineering, Princess Nourah bint Abdulrahman University, Riyadh 11671, Saudi Arabia; 5 Electrical Department, College of Engineering, Alexandria Higher Institute of Engineering and Technology, Alexandria 21421, Egypt

**Keywords:** antenna arrays, microstrip antennas, broadband antennas, frequency selective surfaces

## Abstract

In contemporary wireless communication systems, the multiple-input and multiple-output systems are extensively utilized due to their enhanced spectral efficiency and diversity. Densely packed antenna arrays play an important role in such systems to enhance their spatial diversity, array gain, and beam scanning capabilities. In this article, a slotted meta-material decoupling slab (S-MTM-DS) with dual reflexes slotted E-shapes and an inductive stub is proposed. Its function was validated when located between two microstrip patch antenna elements to reduce the inter-element spacing, the mutual coupling, the return losses, and manufacturing costs due to size reduction. A prototype is simply fabricated in a volume of 67.41 × 33.49 × 1.6 mm^3^ and frequency-span measured from 8.4:11 GHz. At 9.4 GHz frequency, the spaces between the transmitting elements are decreased to 0.57 of the free space wavelength. When the proposed isolation S-MTM-DS is applied, the average isolation among them is measured to be −36 dB, the operational bandwidth is enhanced to be 1.512 GHz, the fractional bandwidth improved to be 16.04%, and the return losses are decreased to be −26.5 dB at 9.4 GHz center frequency. Consequently, the proposed design has the potential to be implemented simply in wireless contemporary communication schemes.

## 1. Introduction

A compact printed Multiple Input Multiple Output (MIMO) antenna is used in wireless communication and radar application, especially in the ultra-wideband waveform transmission application. It has the advantage of having multiple transmission and reception channels that magnifies the need of it [1]. The MIMO antenna may be a single band MIMO antenna such as a USB dongle MIMO and mobile handset antenna system. In addition, it can be a multi-band MIMO such as multi-band mobile handset and tablet PC MIMO antenna system [2]. The advantages of using a MIMO antenna are increasing the channel capacity, improving the spectrum efficiency, and gaining a more reliable network [3]. The MIMO antenna can be easily fabricated and integrated with small communication systems such as cell phones. For example, an eight MIMO antenna operating at a frequency band from 2.6 to 3.5 GHz was fabricated for 5G mobile applications [4]. One of the methods that increases the channel capacity in the 5G mobile systems is to use a massive MIMO antenna. The large number of the receiving and transmitting elements increases the spectral efficiency and reduces the inter-cell interference [2].

Although the MIMO antenna design and implementation are simpler than the array of antenna fabrication, the cost of simplicity is paid in multipath propagation problems and the mutual coupling degradation [5]. Mutual coupling is the effect of not isolating the transmitting or receiving elements from each other. It degrades the angle of arrival estimation and increases the signal to interference noise, which has an adverse effect on the channel capacity and bit error rate in digital transmission and reception systems [5]. Mutual coupling happens due to interaction between the system elements or different radiation in free space on surface tracks. Therefore, the problem of massive MIMO antenna fabrication with low mutual coupling is very important and shall be considered [6].

The mutual coupling in a MIMO antenna can be reduced by minimizing the surface current flow using decoupling networks, etched parasitic elements, split rings resonators, electromagnetic bandgap structures, and defected ground structures [5]. Another method to reduce the mutual coupling is using dielectric resonator antenna or meta-materials [7]. A brief study on the mutual coupling effect on MIMO antenna is in [5,8,9]. The meta-material decoupling slab (MTM-DS) was used between antenna elements to reduce the effect of the mutual coupling. In 9–11 GHz frequency band, the average isolation between elements improved 11 dB with an average gain of around 5 dBi [10]. MTM-DS beat the other decoupling methods in enhancing the undesired front-to-back beam ratio and it is simple to be implemented [11].

In this article, a slotted meta-material decoupling slab (S-MTM-DS) with dual reflexes slotted E-shapes extended with an inductive stub is proposed. Its function was validated when located between two microstrip patch antenna elements to reduce the inter-element spacing, the mutual coupling, the return losses, and manufacturing costs due to size reduction. A prototype is simply fabricated in a volume of FR4 substrate material and the frequency-span is measured in the band 8.4:11 GHz. At 10 GHz frequency, the spaces between each transmitting elements are decreased to 0.57 λ0. When the proposed isolation S-MM-DS is applied, the average isolation among them is measured to be −36 dB, the operational bandwidth is enhanced to 1.512 GHz, the fractional bandwidth improved to 16.04%, and the return losses are decreased to −26.5 dB at a center frequency of 9.4 GHz. The design is simple and evacuates the disadvantage of inferior front-to-back proportion, which is recently announced in other decoupling methods. Therefore, this design is suitable for multiple applications that require stringent execution necessities.

The paper is organized in the following manner: Section 2 is the methodology of design and the measurements analysis. Section 3 presents the fabrication and validation measurements. Section 4 demonstrates the simulation results compared with related works. Finally, Section 5 is the conclusion.

## 2. Methodology of Design and Measurement Analysis

In this section, the coupling conduct of the array components is investigated in detail. The two sorts of coupling phenomenon are: the surface wave phenomena that is limited inside the substrate, and the space wave phenomena that is identified with the near-field or the reactive field coupling and is confined outside the substrate over the coupled patches. The coupling conduct is explored for three distinct arrangements, which are without Decoupling Slab (DS), with Meta-material DS (MM-DS), and with Slotted MM_DS (S-MM-DS).

### 2.1. Antenna Array without Decoupling Slab

**Figure 1:**
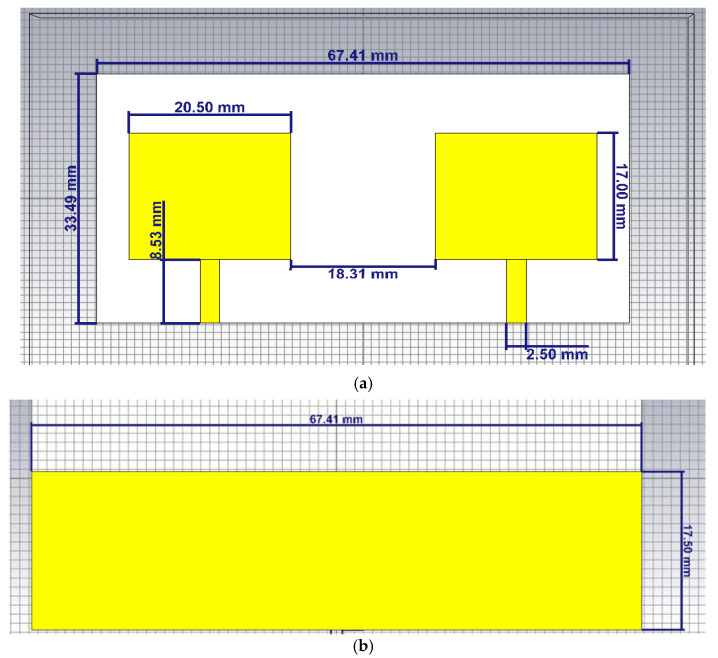
Structure of 2×1 microstrip patch array of antenna without decoupling slab (**a**) front view (**b**) back view.

Utilizing CST Microwave studio, Figure 1a shows a 2 × 1 microstrip patch without DS that comprises one cell of the array of antenna. The impedance bandwidth of the two elements is enhanced by truncating the ground plane as shown in Figure 1b. The dimensions of the two same patch antennas are: L = 17 mm, W = 20.5 mm, and the gap between the two radiators antenna is 18.31 mm. The design will be implemented on FR-4 lossy substrate with thickness (h) equal to 1.6 mm, dielectric constant ($\varepsilon_{r}$) equal to 4.5, and tangent loss (tan δ) equal to 0.025.

**Figure 2:**
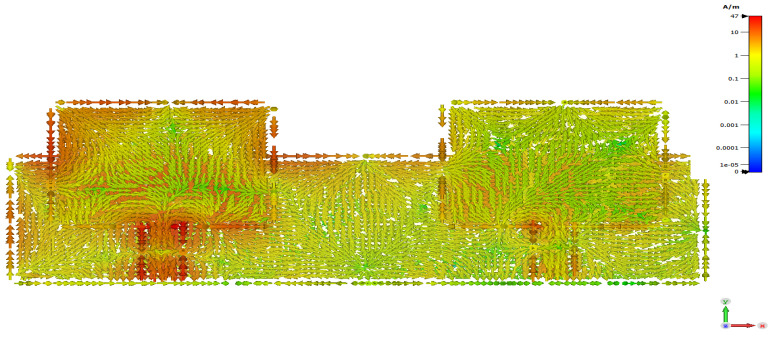
Surface current density of 2×1 microstrip patch antenna array without decoupling slab.

It was found that the current density distributed over the antenna array surface is higher without DS. Figure 2 shows high surface current density.

**Figure 3:**
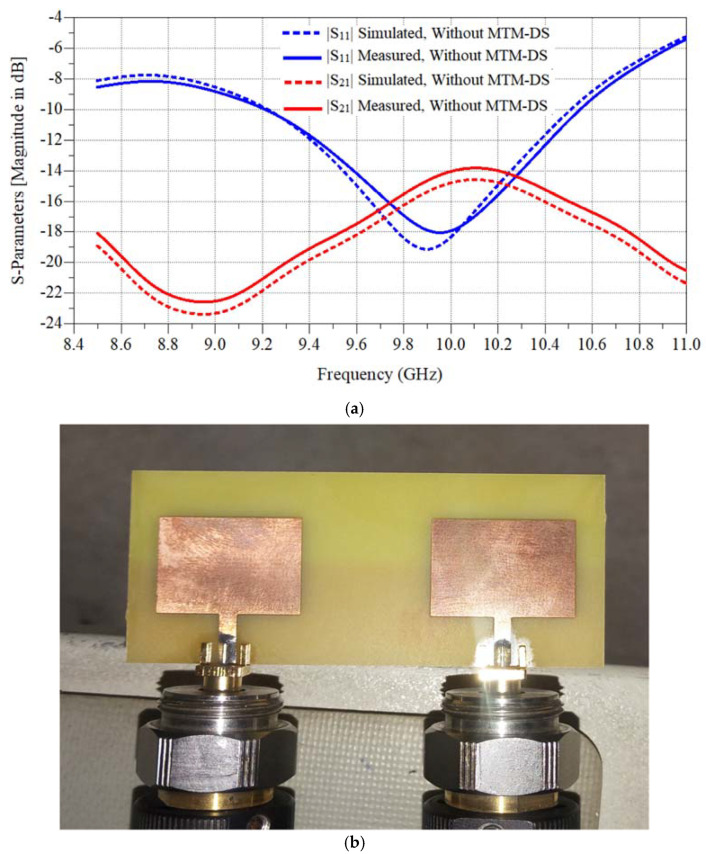
The fabricated 2×1 antenna microstrip patch antenna array without decoupling slab. (**a**) Simulated and measured S-parameters results. (**b**) Array photo.

A computer aid design tool, which is CST Microwave studio, was used to calculate the simulation results. Figure 3a shows the maximum isolation is −23.7 dB and the operating frequency is from 9.22:10.5 GHz with a bandwidth equal to 1.28 GHz. Figure 3b shows a real photo for the fabricated antenna array.

It was assumed in the simulation that the external conditions are absent, that the boundary absorber material is perfectly matched and supporting the concept of putting the array in open space.

### 2.2. Antenna Array with Metamaterial Decoupling Slab

**Figure 4:**
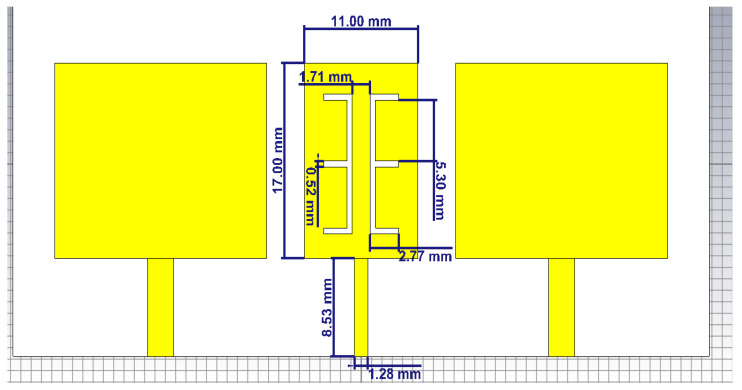
Geometry of 2×1 microstrip patch antenna array with MTM-DS.

The slotted patch antenna was fabricated using meta-material with negative permittivity and permeability [12,13,14]. It was constructed by using 2 E-shaped slits etched in a rectangular microstrip patch. The patch has an open circuited stub with a high impedance at the bottom. The two E-shaped slits are designed to be at an identical distance from a vertical axis at the center of the distance between them [15]. The capacitive nature of the E-shaped slit and the inductance of ¼ wavelength impedance stub can be used in the slotted E-shaped metamaterial decoupling slab for densely packed MIMO antenna. Figure 4 shows the design of the MTM-DS that is proposed in this research. Dimensions are given in Figure 4 and each patch is individually fed by a microstrip feedline.

**Figure 5:**
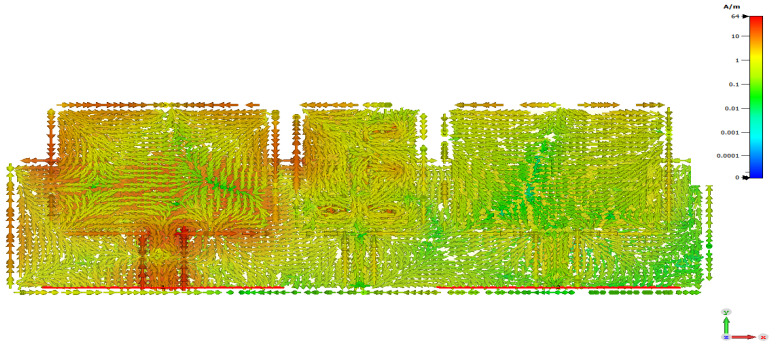
Current density of 2×1 microstrip patch antenna array with MTM-DS.

Figure 5 shows the isolation improves with MTM-DS, which limits the mutual coupling between the two radiators. MTM-DS was implemented on FR-4 lossy substrate with h = 1.6 mm, $\varepsilon_{r}$ = 4.5, and tan δ = 0.025.

**Figure 6:**
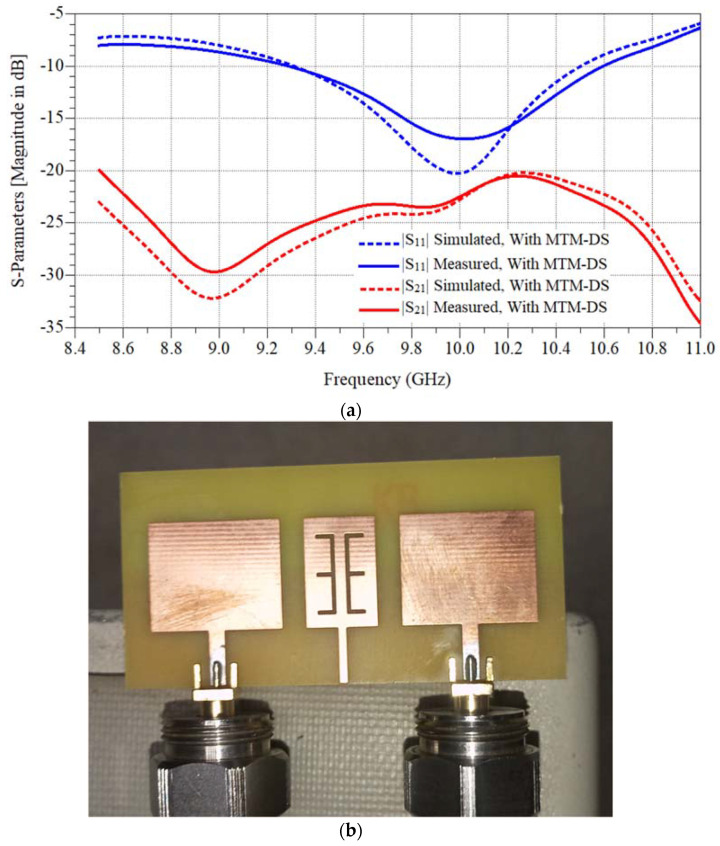
The fabricated 2×1 antenna microstrip patch antenna array with MTM-DS. (**a**) Simulated and measured S-parameters results. (**b**) Connected antenna array photo with decoupling slab.

Figure 6a shows the simulated and measurements parameters of the MTM-DS antenna array. The isolation is −27.5 dB and the operating frequency is from 9.3:10.5 GHz with a bandwidth equal to 1.2 GHz. Figure 6b shows the fabricated array of antenna.

### 2.3. Antenna Array with Slotted Meta-Material Decoupling Slab

**Figure 7:**
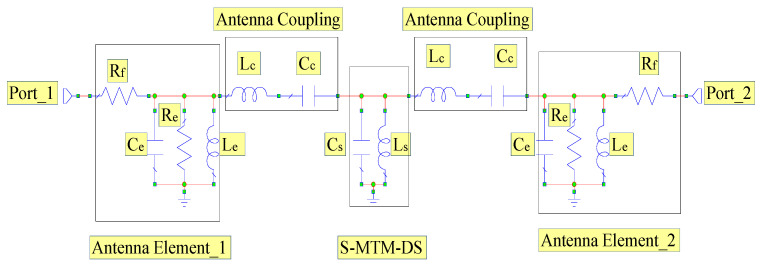
Slotted MTM-DS antenna array equivalent circuit.

A slotted meta-material decoupling slab (S-MTM-DS) with dual reflexes, slotted E-shapes extended with an inductive stub is proposed. Its function was validated when located between two microstrip patch antenna elements to reduce the inter-element spacing, the mutual coupling, and the return losses. Figure 7 shows the slotted MTM-DS antenna array equivalent circuit. The radiator is presented in the equivalent circuit by a resonant circuit with resistance *R_e_*, capacitance *C_e_*, and inductance *L_e_*. The slotted MTM-DS has inductance *L_s_* and the capacitance *C_s_*.

Slotted MTM-DS in the middle of the array connecting the two antenna elements sections is modelled by inductance *L_c_*. The coupling between the Slotted MTM-DS and the patch is through the dominant capacitance *C_c_* because the coupling between the patch and the Slotted MTM-DS is via the patch antenna non-radiating edge. The resonance frequency ($f_{r}$) of the slotted MTM-DS is dependent on the magnitude of *L_c_* and *C_s_* and is presented as follows:

(1) $$f_{r}=\frac{1}{2\pi \sqrt{L_{s}C_{s}}}$$

The effectiveness of the slotted MTM-DS was determined by the simplified equivalent circuit model. Optimized values of the equivalent circuit model were determined by using an optimization tool in full-wave electromagnetic solver simulation by AWR. The magnitudes of these parameters are given in Table 1.

**Figure 8:**
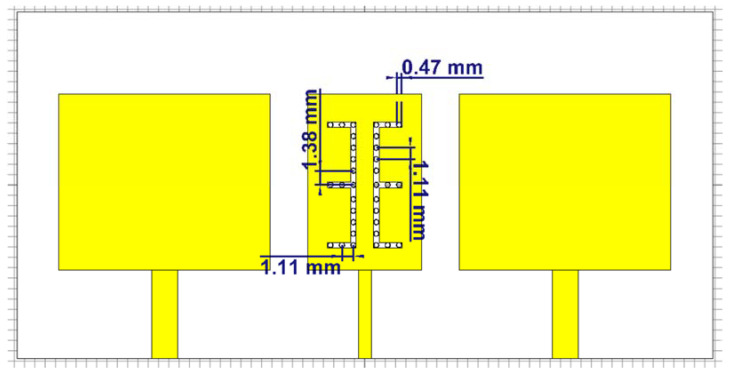
Geometry of 2×1 microstrip patch antenna array with slotted MTM-DS.

From the previous simulations and measurements, it was necessary to insert the slotted MTM-DS between the two radiators as shown in Figure 8 to enhance the mutual coupling. The MTM-DS microstrip configuration has ground slots on each arm. By using this configuration, the gain and the bandwidth of the array of elements is not affected by the presence of the slots.

**Figure 9:**
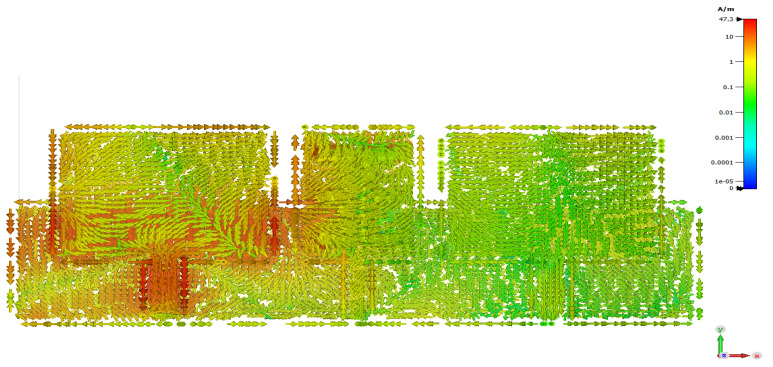
Current density of 2×1 microstrip patch antenna array with slotted MTM-DS.

The importance of the new designed slotted MTM-DS appears in suppressing the induced surface current that results from interaction between the two patches. It is observable in Figure 9 that strong current is induced on the array of the antenna that guarantees the effectiveness of the slotted MTM-DS. The distance between the two patch elements is 0.57λ0, where λ0 is wavelength in free space at 9.4 GHz.

**Figure 10:**
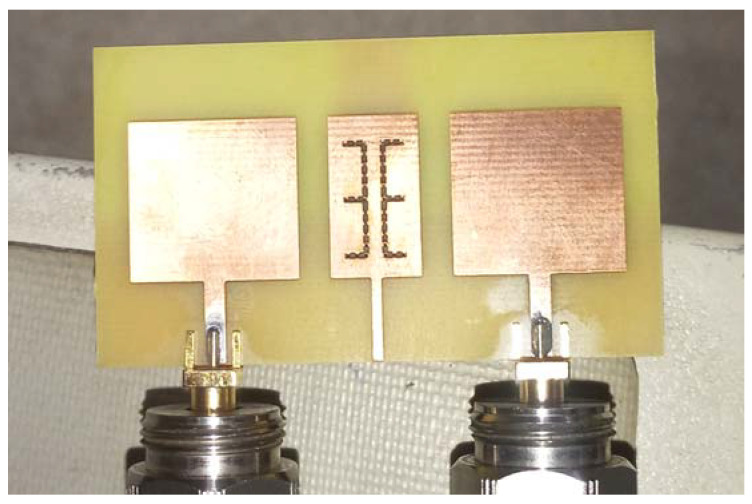
Photograph of the fabricated antenna array with slotted MTM-DS.

**Figure 11:**
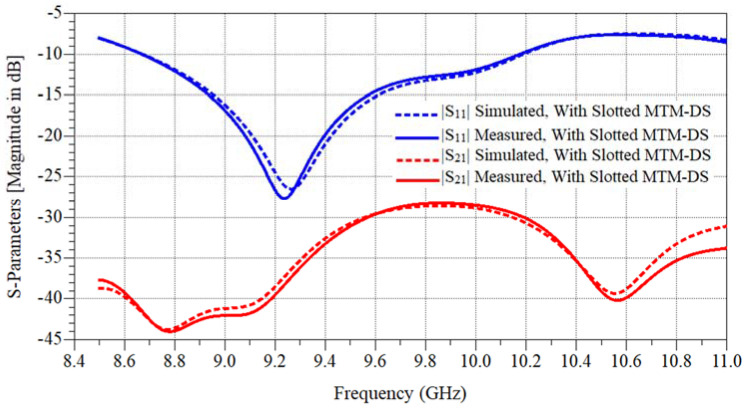
Simulated and measured S-parameters results of the fabricated 2 × 1 antenna microstrip patch antenna array with slotted MTM-DS.

MTM-DS was designed and implemented as previous on FR-4 lossy substrate with h = 1.6 mm, $\varepsilon_{r}$ = 4.5, and tan δ = 0.025 as shown in Figure 10. The simulated and measured results isolation and return loss response of the MTM-DS array of antenna is shown in Figure 11. In Figure 11 the frequency bandwidth is 1.51 GHz from 8.67:10.18 GHz, and the maximum isolation is −43.7 dB.

S-parameter results are measured the three types of the array of antenna: MTM without DS, MTM-DS, and slotted MTM-DS are summarized in Table 2. For the first configuration (without DS), the average isolation over the proposed band width is −18 dB. For the second (MTM-DS), it is −23.85 dB, and for the last one (slotted MTM-DS), it is −36 dB. On average the isolation is improved by 18 dB.

## 3. Radiation Pattern of the Antenna Arrays

**Figure 12:**
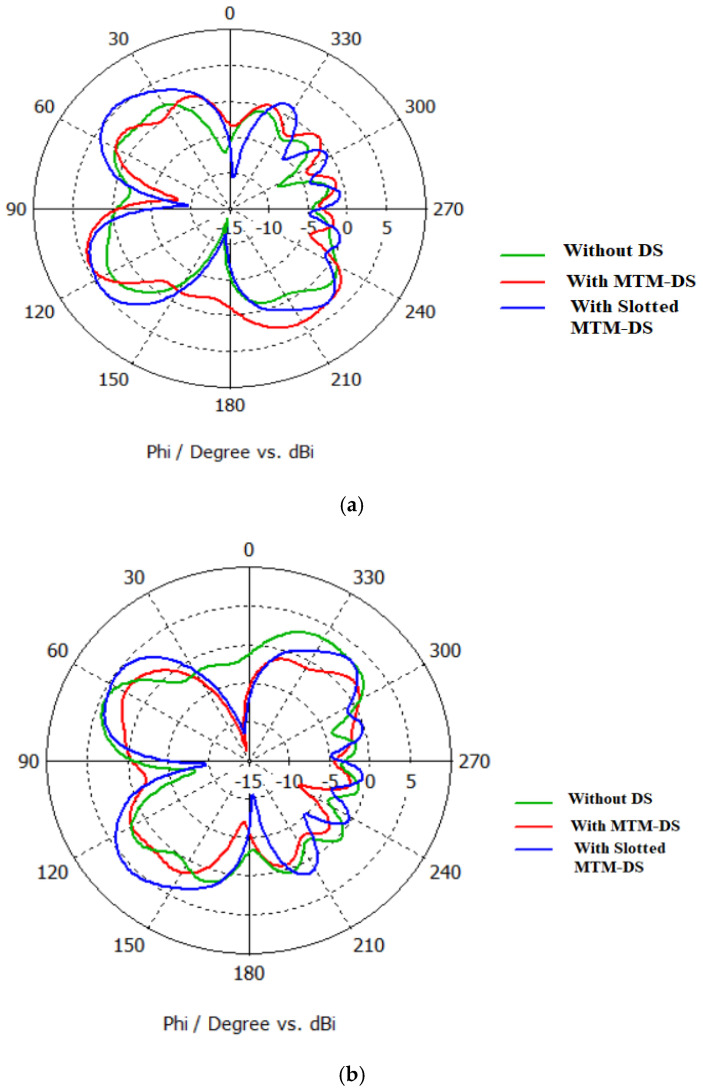
Simulated polar plots of the antenna array without DS, with MTM-DS, and with slotted MTM-DS (**a**) Antenna element #1 (**b**) Antenna element #2.

The simulated 2D polar plots of the three types of the array of antenna: MTM without DS, MTM-DS, and slotted MTM-DS at 9.5 GHz are shown in Figure 12. A good pattern correlation in 2D is observed for the three types of the antenna array. Although the electric plane for the three types is slightly the same, the magnetic plane for the slotted MTM-DS is minimal, and the gain is improved at the selected frequency 9.5 GHz.

## 4. Results and Discussion

The proposed algorithm is compared with previously published research in designing and implementing the array of antennas in the same frequency band. Table 3 shows the measurements results of three parameters: maximum isolation, patch separation, operating bandwidth reduction, design complexity, and cost for the three isolating techniques discussed in this work. A merit was adjusted for each parameter where the best value takes 100% and the worst value take (worst/best) × 100. The average percentage was calculated for each technique. The average percentage for the complementary split-ring resonators [16] is the best (88%); however, the design complexity and the cost are high, which makes it an unprovable solution for the design and implementation problem. The second in the rank is both: the complementary split-ring resonator [17] and the meta-material decoupling slab [18]. In [17], the maximum isolation was low compared to the slotted MTM-DS, and in [15], although the maximum isolation was better than slotted MTM-DS, the average isolation was worse than it. Additionally, the separation between the patches is 0.66 λ0, which is worse than it in the slotted MTM-DS which is 0.57 λ0. Although the presented technique in this research (slotted MTM-DS) has the third rank in the average percentage merit, it outperforms the other three techniques in [16,17], and [18] in the average isolation and the parameter value, design complexity, and cost forms a wonderful assembly to design and implement easily.

## 5. Conclusions

A proficient method of including S-MM-DS is proposed for mutual coupling concealment in dense arrays with edge partition of 0.575 λ0. The S-MTM-DS structure was carved, drilled, and plated on a section between the radiating elements. The S-MM-DS is equipped for representing mutual coupling brought about by surface waves just as space waves. For 1.6 thick and 4.5 permittivity substrate the mutual coupling decrease of −43.7 dB was accomplished with the decoupling slab set between array components. The structure is effectively feasible and can be utilized in all respects viably in beam scanning applications. The real advantage of this plan is that it tends to be effectively created and can be strategically located where the mutual coupling concealment is wanted. The S-MTM-DS is created from a similar material as that of exhibit components consequently making this system exceptionally adaptable in wording of its advantages and applications.

## Figures and Tables

**Table 1:** Optimized values of the equivalent model representing the 2 × 1 antenna microstrip patch antenna array with slotted MTM-DS.

Parameter	Values
*R_f_*	54 Ohm
*R_e_*	1.9 Ohm
*C_e_*	0.65 pF
*L_e_*	4.29 nH
*L_c_*	0.18 nH
*C_c_*	2.66 pF
*L_s_*	0.68 nH
*C_s_*	4.05 pF

**Table 2:** Measurements resulted from the three isolating techniques.

Case	Operation BW (GHz)	BW (GHz) S11 < 10 dB	Fraction BW (%)	Mutual Coupling Suppression between Antenna Array(dB)		
				Min	Ave	Max
Without DS	9.22–10.5	1.28	12.98	−14.5	−18	−23.7
With MTM-DS	9.3–10.5	1.2	12.12	−20.2	−23.8	−27.5
With slotted MTM-DS	8.67–10.18	1.51	16.08	−28.5	−36	−43.7

**Table 3:** Measurements resulted from the Three Isolating Techniques against previously presented work in the literature.

Ref	Method	Max. Isolation Improvement	Patch Separation (λ0)	Operating Bandwidth Reduction (%)	Average Percentage	Design Complexity	Cost
[19]	Shorted annular elliptical patch	8 (14%)	0.75 (16%)	81%	37%	Moderate	Medium
[20]	Ring of magnetic current	10 (18%)	0.5 (25%)	87%	40%	Moderate	Medium
[16]	Complementary split-ring resonators	37 (65%)	0.125 (100%)	100%	88%	High	High
[21]	meta-surface wall isolator	13.5 (24%)	1.16 (11%)	100%	45%	Low	Low
[17]	Complementary split-ring resonator	27 (47%)	0.125 (100%)	71%	73%	Low	Low
[22]	U-shaped microstrip line	17 (30%)	0.75 (16%)	88%	45%	Moderate	Medium
[23]	Periodically grounded edge-coupled split-ring resonators	18 (32%)	0.5 (25%)	100%	52%	High	High
[18]	meta-material decoupling slab	57 (100%)	0.66 (19%)	100%	73%	Low	Low
Proposed Work	slotted meta-material decoupling slab	43.7 (76%)	0.57 (22%)	100%	66%	Moderate	Low
